# Patient-based quality control for glucometers: using the moving sum of positive patient results and moving average

**DOI:** 10.11613/BM.2020.020709

**Published:** 2020-06-15

**Authors:** Chun Yee Lim, Tony Badrick, Tze Ping Loh

**Affiliations:** 1 Engineering Cluster, Singapore Institute of Technology, Singapore; 2 RCPA Quality Assurance Programs, St Leonards, Sydney, Australia; 3 International Federation of Clinical Chemistry and Laboratory Medicine Committee on Analytical Quality; 4 Department of Laboratory Medicine, National University Hospital, Singapore

**Keywords:** laboratory management, quality control, moving average, moving sum of positive patient results, patient-based quality control

## Abstract

**Introduction:**

The capability of glucometer internal quality control (QC) in detecting varying magnitude of systematic error (bias), and the potential use of moving sum of positive results (MovSum) and moving average (MA) techniques as potential alternatives were evaluated.

**Materials and methods:**

The probability of error detection using routine QC and manufacturer’s control limits were investigated using historical data. Moving sum of positive results and MA algorithms were developed and optimized before being evaluated through numerical simulation for false positive rate and probability of error detection.

**Results:**

When the manufacturer’s default control limits (that are multiple times higher than the running standard deviation (SD) of the glucometer) was used, they had 0-75% probability of detecting small errors up to 0.8 mmol/L. However, the error detection capability improved to 20-100% when the running SD of the glucometer was used. At a binarization threshold of 6.2 mmol/L and block sizes of 200 to 400, MovSum has a 100% probability of detecting a bias that is greater than 0.5 mmol/L. Compared to MovSum, the MA technique had lower probability of bias detection, especially for smaller bias magnitudes; MA also had higher false positive rates.

**Conclusions:**

The MovSum technique is suited for detecting small, but clinically significant biases. Point of care QC should follow conventional practice by setting the control limits according to the running mean and SD to allow proper error detection. The glucometer manufacturers have an active role to play in liberalizing QC settings and also enhancing the middleware to facility patient-based QC practices.

## Introduction

Point of care glucose meter (glucometer) is the most commonly used point of care laboratory device in the hospital. In some settings, glucometers can generate nearly half of the clinical biochemistry laboratory results. Point of care glucose testing is generally performed by non-laboratory staff to monitor short-term glycemic control and detect hypoglycaemic and hyperglycaemic crisis (*1*, *2*). Consequently, abnormal results are often acted upon quickly. The widespread use of ward- and clinic-based glucometers has raised the awareness and requirement to ensure the quality of the results generated are comparable (although not equivalent) to the core laboratory methods (*2*).

Ensuring this result harmonization can be achieved by performing pre-deployment instrument evaluation, relevant training and ongoing competency certification of performing staff, establishing an internal quality control (QC) and proficiency testing system, and having laboratory oversight (*1*-*4*). The QC testing on glucometer is performed using at least two concentrations of reference materials provided by the manufacturer and compared against the recommended target ranges. This testing is commonly performed once a day by a key operator (*4*). The QC acceptability range provided by the manufacturers is often very wide to minimise false error flags. Such wide control limits are likely to miss smaller biases that may be clinically important. Moreover, the QC material provided by the manufacturer is not commutable to the whole blood sample type used in clinical settings.

Patient-based real-time quality control techniques, such as moving average (MA), have received increasing attention in the routine laboratory as a powerful tool to improve error detection and overcome the non-commutability issue present with many reference materials (*5*, *6*). The moving sum of positive results (MovSum) method has been previously described to be able to detect very low positive bias and imprecision in assays (*7*, *8*). However, its use in the point of care setting remains under-explored, largely because of the small numbers of samples run on a particular glucometer and the batch mode of analysis inherent in this type of testing. In this work, we evaluated the capability of glucometer QC in detecting varying magnitude of systematic error (bias) and investigated the potential use of MA and MovSum techniques as alternatives.

## Materials and methods

This study was performed at the National University Hospital, Singapore, between October and December 2019. The data extraction period was from November 2014 to July 2015.

### Capability of internal quality control in detecting systematic error

The Accu-Chek Inform II system (Roche Diagnostics, Mannheim, Germany) is a glucometer that is widely used in the hospital setting. The QC target range provided by the manufacturer and preprogrammed into the devices were: level 1 (target: 2.5 mmol/L, range: 1.7-3.3 mmol/L) and level 2 (target: 17.0 mmol/L, range: 14.5-19.5 mmol/L). The actual analytical standard deviation (SD) of these reference materials derived from one month of QC measurements were: level 1 (mean: 2.4 mmol/L, SD: 0.075 mmol/L, CV: 3.1%); level 2 (mean 16.8 mmol/L, SD: 0.37 mmol/L, CV: 2.2%).

To investigate the effect of using such wide control limits, we calculated the probability of error detection using z = [N of SD used in the control limit - (systematic error/analytical SD)] (*8*). For example, a positive bias detection at level 1 QC requires the triggering of the 1:13S rule, since the default upper control limit was 13 times the running analytical SD. When a systematic bias is introduced, the z-value which triggers the 1:13S QC rule is given by:



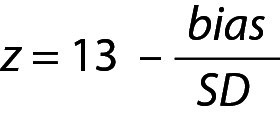



Assume that there is a bias of +0.8 mmol/L introduced, then we have from the equation above that,
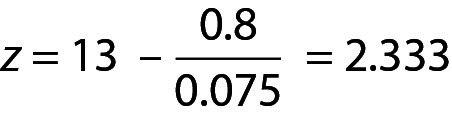
and the corresponding P value for detection of the bias = 0.0098 or 0.98% using Z-table.

On the other hand, the probability of error detection for various QC rules for a given bias, if the user was able to set the control limits according to the actual running SD, are shown below.

1:3S rule

For example, a positive bias detection at level 2 QC with the running SD of the glucometer requires the triggering of the 1:3S. When a positive has is introduced, the z-value which triggers the 1:3S QC rule is given by:



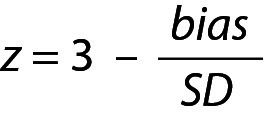



For level 2 QC with a bias of +0.3 mmol/L,



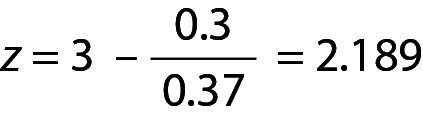



the corresponding P value for detection of the bias = 0.014 or 1.4% using Z-table.

2:2S rule

Similarly, the probability of the small positive bias of + 0.3 mmol/L to be picked up by the 2:2S QC rule can be calculated as follows:

For a single point to lie outside of 2S zone:
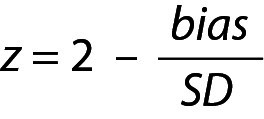


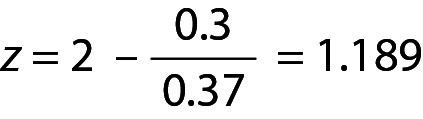
the corresponding P value for detection of the bias = 0.117 or 11.7% using Z-table. Hence, the chance of 2 consecutive points lying outside of the 2S zone = 0.117 × 0.117 = 0.0137 or 1.4%. The corresponding probabilities of detecting varying magnitudes of bias ranging from 0.1-2.5 mmol/L were further evaluated.

### Bias detection with moving sum of positives (MovSum)

Ten months of de-identified results from a glucometer (N = 12,334) belonging to a general medicine ward were extracted. It was assumed that the glucometer did not suffer from a systematic error during this period (no QC failure). The results exhibits a typical log-normal distribution.

**Figure 1:**
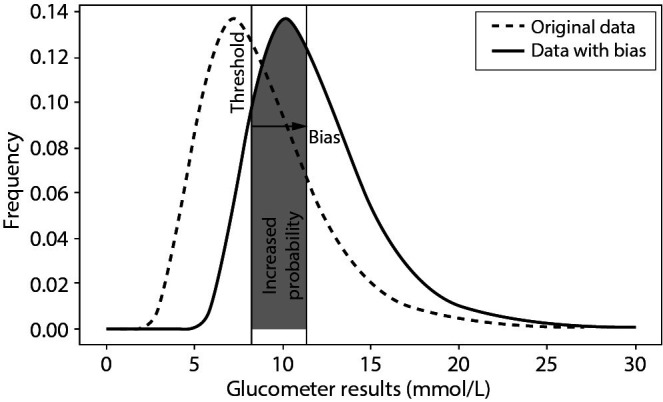
Shift of glucometer results distribution due to bias.

The key concept of this technique is setting a binarization threshold (T) for quantitative laboratory results and classifying them as a positive (assigned as “1”) or negative (assigned as “0”) statuses. A moving block of results is then included for summation of the positive results, *i.e*., the sum of the results that have to be binarised and converted as “1”. The block size, N, is the number of results included in the summation. Each time a new result is available, it is recruited into the block while the oldest result is removed, and the total number of positive results is recalculated - hence, it is a moving sum. If a persistent bias is introduced into the analytical system, the entire population will shift. This shift will lead to an increased proportion of results being classified as positive or negative (Figure 1). The MovSum will increase and eventually exceed the predefined control limits.

To minimize the false positive rates, the upper and lower control limits were set as the highest and lowest MovSum values in the original data set without any artificial bias introduction. A bias is detected when the MovSum value moves beyond or equal to the control limits. The inclusion of upper and lower control limits in the triggering of bias detection is intentional as it was found to improve the bias detection performance significantly at the expense of a non-zero but small false positive rate. The impact of the two other parameters (binarization threshold, T and block size, N) on the error detection capability was evaluated. This simulation was performed by artificially introducing sustained positive and negative bias ranging from 0.5 to 3 mmol/L into various points in the original data set at every 50^th^ data points, up to a total of 200 separate runs. The first 400 data points are excluded from the bias introduction to allow sufficient data points for the moving sum calculation. Binarization thresholds ranging from 5.0-9.0 mmol/L were examined. For each T, block sizes of 100-400 were evaluated.

The error detection capabilities were measured in terms of the median number of patients affected before error detection (MNPed, averaged over the 200 runs), bias detection rate (the probability of error detection out of the 200 runs). At the same time, the false positive rates of the different parameter permutations were also recorded.

### Bias detection with moving average

The same data set was also subjected to the MA examination. Details of setting up MA has been well described (*5*, *6*). The MA approach algorithm calculates the average within a moving block of patient results. Each time a new result is available, it is incorporated into the block and the earliest result is discarded (*i.e*., the block ‘moves’ forward by one result a time). The average of the new block is recalculated and compared to predefined control limits. Truncation limits are applied to minimize the impact of extreme results on the calculation of the MA. The bias introduction, measurement of bias detection capability, and false positive rates were performed as the preceding section for MovSum. The initial parameters for the MA set up were based on those previously described by Huub et al. for serum glucose measurements (*9*). They were: truncation limit of less than or equal to 10 mmol/L, control limits of 5.1 mmol/L and 6.85 mmol/L and a block size of 25. Block sizes of 25, 30, 40, 50, and 80 were further evaluated in this study. The control limits were also adjusted to between 5.4 and 8.6 mmol/L to reduce the false alarm rate and improve bias detection.

### Statistical analysis

All data analyses and simulations were performed with Python 3 (Jupyter Notebook, https://jupyter.org/install) and standard libraries including NumPy (https://numpy.org/), Matplotlib (https://matplotlib.org/) and Pandas (https://pandas.pydata.org/) on an HP Z440 workstation (Intel Xeon CPU E5-1650v4).

## Results

### Capability of internal quality control in detecting systematic error

Based on the historical QC data, the manufacturer’s present acceptable QC limits are 6-13 times larger than the actual running analytical SD (Table 1). The probability of QC detection at the varying magnitude of the bias is summarised in Table 2. When the QC range provided by the manufacturer was used as the control limits, they had 0-75% probability of detecting small errors up to 0.8 mmol/L. The asymmetry in the probability of error detection between the upper and lower control limit is due to the difference between the QC target and the mean of the running QC measurement, as would be present in a real-world scenario. However, when QC target and control limits are set according to the running mean and SD of the QC measurements, the probability of error detection improved significantly with the level 1 QC, detecting all biases 0.5 mmol/L and larger for the 3 QC rules examined.

**Table 1:** Glucometer internal quality control, with the upper and lower control limits given by the default device manufacturer’s settings

**QC level**	**Upper control limit**	**Lower control limit**	**Mean **	**SD**	**CV_a_**	**Number of SD away from mean to trigger QC violation**	
						**Upper control limit**	**Lower control limit**
1	3.3	1.7	2.4	0.08	3.1%	~ 13	~ 10
2	19.6	14.5	16.8	0.37	2.2%	~ 8	~ 6
The mean, standard deviation (SD) and coefficient of variation (CVa) are derived from 1 month’s running QC results of the glucometer. All values are in mmol/L. QC - quality control.							

**Table 2:** The probability of internal quality control in detecting varying magnitude of bias using different control limits

**UCL and LCL according to glucometer manufacturer’s default setting**									
		**Bias magnitude (mmol/L)**							
	**QC Rule**	**0.1**	**0.3**	**0.5**	**0.8**	**1**	**1.5**	**2**	**2.5**
**Level 1 QC, 2.4 mmol/L**	LCL - 1:10S	0%	0%	0.043%	75%	100%	100%	100%	100%
	UCL - 1:13S	0%	0%	0%	0.98%	63%	100%	100%	100%
**Level 2 QC, 16.8 mmol/L**	LCL - 1:6S	0%	0%	0%	0%	0.049%	2.6%	28%	78%
	UCL - 1:8S	0%	0%	0%	0%	0%	0%	0.47%	11%
**UCL and LCL set according to the running SD of the glucometer**									
**Level 1 QC, 2.4 mmol/L**	1:3S	4.8%	84%	100%	100%	100%	100%	100%	100%
	2:2S	6.4%	96%	100%	100%	100%	100%	100%	100%
	4:1S	16%	100%	100%	100%	100%	100%	100%	100%
**Level 2 QC, 16.8 mmol/L**	1:3S	0.32%	1.4%	5.0%	20%	38%	85%	99%	100%
	2:2S	0.18%	1.4%	6.7%	32%	58%	96%	100%	100%
	4:1S	0.29%	3.3%	16%	59%	83%	100%	100%	100%
UCL - upper control limit. LCL - lower control limit. QC - quality control. SD - standard deviation.									

### Bias detection with moving sum of positives

**Figure 2:**
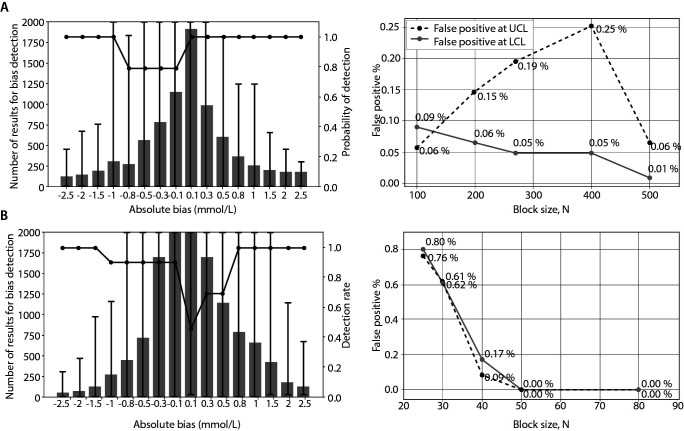
Performance of (A) the moving sum of positive results method (threshold, *T* and block size, *N* are set to 6.2 mmol/L and 270 respectively) and (B) the moving average method (*N* is set to 40 and truncation limit is ≤ 10.0 mmol/L while the upper control limit, UCL and the lower control limit, LCL are set to 8.6 mmol/L and 5.4 mmol/L respectively) for detection of absolute bias ranging from 0.1 to 2.5 mmol/L. The bar charts on the left column show the median number of results for bias detection with the upper and lower whiskers showing the maximum and minimum values, respectively. The line charts overlaid in the bar chart show the probability of detection. The chart on the right columns shows the false positive rate for various *N* for both methods.

**Figure 3:**
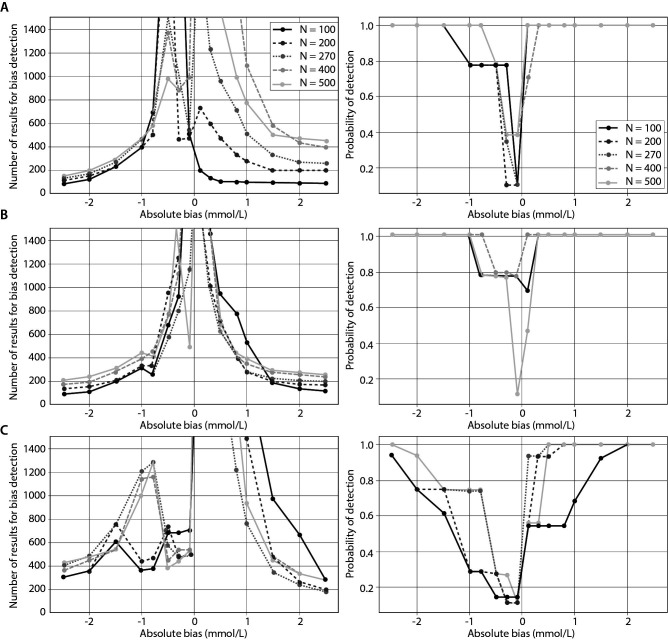
Performance of the moving sum of positive results method expressed as number of results for bias detection (left column) and probability of detection (right column) when binarization threshold, *T* is set to (A) 5.0 mmol/L, (B) 6.2 mmol/L and (C) 8.3 mmol/L as block size, *N* is varied from 100 to 500.

**Figure 4:**
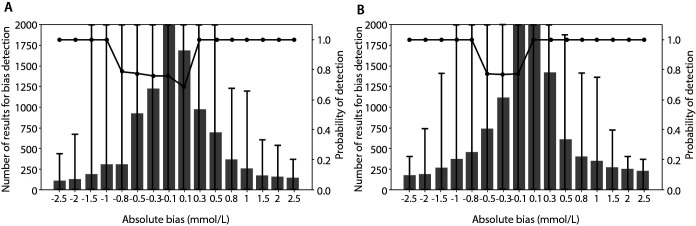
Performance of the moving sum of positive results method for detection of absolute bias ranging from 0.1 to 2.5 mmol/L. The bar chart shows the median number of results for bias detection with the upper and lower whiskers showing the maximum and minimum values, respectively. The line charts overlaid in each figure show the probability of detection. Threshold, *T* is held at 6.2 mmol/L while block size, *N* is set to (A) 200 (B) 400.

The extracted laboratory data showed: a mean of 8.9 mmol/L, a median of 8.2 mmol/L, mode of 6.7 mmol/L. Control limits of the MovSum algorithm for selected T and N, set as the max and min of the moving sum for the training data set without the artificial introduction of bias, are summarised in Table 3. The bias detection capabilities and false positive rates of selected T and N were summarized in Table 4, and graphically presented in Figures 2 and 3 to illustrate their impact. Among the permutations of parameters selected in this simulation, the best T was 6.2 mmol/L with N of 200 to 400 (Figures 2 and 4). A smaller block size of 200 or 270 demonstrated a smaller MNPed and detected the biases faster while a larger block size of 400 yields an overall higher detection rate (larger than 77% for all bias magnitudes investigated).

**Table 3:** Control limits of the moving sum of positive results, set as the maximum and minimum of the moving sum values for the training data set without the artificial introduction of bias.

**Block size, N**	**Upper control limit**			**Lower control limit**		
**Binarization threshold = 5.0 mmol/L**	**Binarization threshold = 6.2 mmol/L**	**Binarization threshold = 8.3 mmol/L**	**Binarization threshold = 5.0 mmol/L**	**Binarization threshold = 6.2 mmol/L**	**Binarization threshold = 8.3 mmol/L**
100	100	100	94	64	48	8
200	200	190	162	146	114	32
270	270	250	198	204	166	48
400	400	362	296	320	256	96
500	496	444	346	414	324	130

**Table 4:** Median number of patient results affected before error detection and probability of error detection for a bias of +0.5 mmol/L with false positive rate of the moving sum of positive results method when binarization threshold is set to 5 mmol/L, 6.2 mmol/L and 8.3 mmol/L for various block sizes.

	**Binarization threshold = 5.0 mmol/L**				**Binarization threshold = 6.2 mmol/L**				**Binarization threshold = 8.3 mmol/L**			
**Block size,** **N**	**Median number of patient results affected before error detection**	**Probability of error detection**	**False positive at UCL**	**False positive at LCL**	**Median number of patient results affected before error detection**	**Probability of error detection**	**False positive at UCL**	**False positive at LCL**	**Median number of patient results affected before error detection**	**Probability of error detection**	**False positive at UCL**	**False positive at LCL**
100	100	100%	14.79%	0.13%	930	100%	0.06%	0.09%	2727	54.5%	1.22%	0.01%
200	466	100%	4.05%	0.01%	695	100%	0.15%	0.06%	2287	93.5%	0.19%	0.05%
270	957	100%	1.22%	0.01%	611	100%	0.19%	0.05%	2128	100%	0.04%	0.07%
400	2207	100%	0.12%	0.02%	604	100%	0.25%	0.05%	2848	100%	0.04%	0.02%
500	1544	100%	0.33%	0.06%	677	100%	0.06%	0.01%	2091	100%	0.18%	0.11%
UCL - upper control limit. LCL - lower control limit.												

### Bias detection with moving average

The truncation limits (TL) are necessary for MA as it is highly susceptible to extreme values, which are common in the use of glucometer in the hospital setting. The optimized block size and control limits were 40 and 5.4-8.6 mmol/L, respectively. At these parameters, the false positive rate was 0.27% (Figure 2). While the MNPed were comparable to the MovSum method, the probability of bias detection was lower, especially for smaller bias magnitudes. For example, the detection rate of a positive bias of +0.3 mmol/L for MA method is 69% with MNPed of 1718 while the detection rate of the same bias with MovSum method is 100%, at a smaller MNPed of 992 and a similar false positive rate of 0.24% (for T = 6.2 mmol/L, N = 270). On the contrary, the MA method is well-suited for the detection of larger bias with lower MNPed. For example, the MNPed of the MA method for the detection of - 1.5 mmol/L was 119 while the MNPed of MovSum method (for T = 6.2 mmol/L, N = 270) was larger, at 197.

## Discussion

This study demonstrated that the control limits set by the glucometer manufacturer are too wide to detect clinically important low concentration biases using routine QC. On the other hand, MovSum is capable of detecting such biases and can potentially be adopted to complement routine QC. Moving average has low error detection capability for low concentration biases when compared to MovSum, and is not an optimal technique for this purpose.

The minimum analytical performance specification for glucometer has been defined differently by different medical professional bodies. For example, College of American Pathologists: 0.3 mmol/L; Royal College of Pathologists Australasia: 0.5 mmol/L; U.S. Food and Drug Administration: 0.7 mmol/L; Institute for Quality Management in Healthcare: 1 mmol/L (*10*, *11*). A bias as small as 0.3 mmol/L could be considered clinically important, particularly for low glucose concentrations because even a small (positive) bias has the potential to mask a hypoglycaemic episode, which is biochemically variably defined as 2.5-3.0 mmol/L (*12*). A missed hypoglycaemia event can have severe clinical consequences, including irreversible neuroglycopenic damage and even death.

From the results of this study, it is apparent that the broad QC target range provided by the manufacturer cannot satisfactorily detect analytical bias < 1 mmol/L, even when a QC material with low glucose concentration is used. Indeed, a probability of detection of 1% for a 1.0 mmol/L positive bias meant that it would take an average of 100 QC testing episodes to detect the error. In most situations, this translates into delayed bias detection for 100 days, assuming QC testing is performed daily. This approach puts patients at significant clinical risk in the interim until the bias is detected. Even when the QC limit is breached, most end-users would repeat another QC measurement, which will most likely fall within the control limit. Moreover, it should also be noted that the difference between the running QC means and the QC target provided by the manufacturer will result in an asymmetrical bias detection capability.

The error detection for QC can be significantly improved by using the running mean and SD to set the QC target and control limits. This practice is particularly true for the low concentration QC, where the probability of detection was 100% for a bias of 0.5 mmol/L. However, it is not always possible to manually input the QC control limits into the glucometers due to instrument software limitations. As such, the performance of the QC needs to be monitored manually using either middleware, laboratory information system or other software. Another major limitation of internal QC practice is the use of non-commutable materials, which cannot be ameliorated by statistical manipulation. Indeed, it is unclear how well QC materials provided by manufacturers behave like whole human blood and whether they can materially pick up errors that affect human sample testing.

The MovSum is a sensitive method for detecting small biases (*8*). At T of 6.2 mmol/L and N of 200 to 400, it has a 100% probability of detecting a bias that is greater than 0.5 mmol/L. Importantly, it can detect all positive biases, which bears the risks of masking a hypoglycaemia diagnosis. Nevertheless, the MNPed is relatively large, on average, affecting 600-700 results before detecting a bias of 0.5 mmol/L. Considering a glucometer that produces 40-50 measurements *per* day, it would take approximately two weeks to detect the bias. This process is significantly better when compared to traditional QC using the acceptability range provided by the manufacturer. It is conceivable that combining the data from multiple glucometers will significantly shorten the time to error detection. Nevertheless, such practice will increase the detection of sources of error that affects all the glucometers (*e.g.*, a compromised lot of test cartridges) while potentially compromising errors that are specific to a glucometer (*e.g.*, faulty sensor) as other functioning glucometers may mask the error.

The MovSum method is relatively easy to set up, requiring only the adjustment of two parameters: T and N. This was previously demonstrated on the prostate-specific antigen assay, where most of the results were very low (*8*). Consequently, the binarization threshold was also set very low. In this study, the binarization thresholds near the central tendencies (mean, mode, median) were explored. A T that is close to the mode of the population is more sensitive to bias since a small change will shift a larger proportion of the population into a different classification. However, if the T was set to a significantly lower than mode (for example 5.0 mmol/L), the false positive rate increased significantly, especially when coupled with a small N. With a small N and T, there is a high probability to find an entire block filled with positive results, even in the absence of any bias, thus violating the UCL and triggering a false alarm. On the contrary, selecting a T which is significantly larger than the mode resulted in a decrease in detection rate and an increase in MNPed, especially when paired with a small N. This observation can be attributed to the fact that, as T is set near the “tail” of the log-normal distribution, a shift in the population will only induce a small proportion of the results to shift classification, resulting in a lower sensitivity.

**Figure 5:**
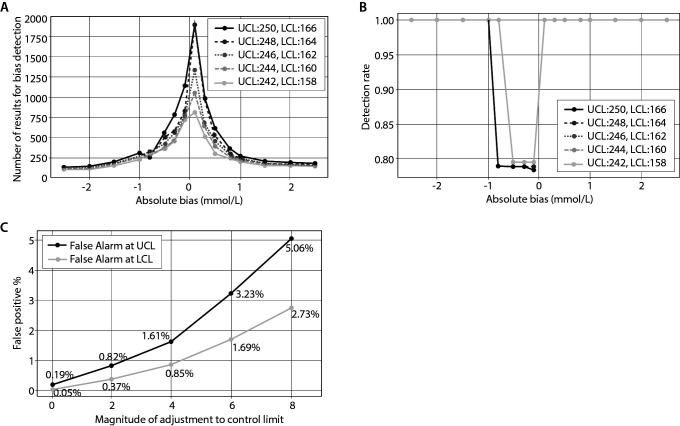
Performance of moving sum of positive results method expressed as (A) number of results for bias detection, (B) probability of detection and (C) false positive percentage when the upper (UCL) and lower control limits (LCL) are tightened. New UCL = (original UCL – adjustment) and new LCL = (original LCL + adjustment). The original UCL and LCL are 250 and 166, respectively. Binarization threshold (T) and block size (N) are held constant at 6.2 mmol/L and 270, respectively.

Using the minimum and maximum value of the MovSum of the training data set as control limits will ensure the lowest false positive rate. The ideal choice of N is typically larger than that of the MA technique because the underlying principle of MovSum method is based on the shift in the data distribution. The control limits can be made narrowed to improve the detection capability (lower MNPed), although the false positive rate will rise (Figure 5). The gain in detection capability must be carefully weighed against the potential unproductive effort and resources expended on troubleshooting a false alarm. In this study, the MA technique was not rigorously optimized, making it difficult to draw a firm conclusion regarding the differences in performance compared to MovSum. However, MA is generally more susceptible to extreme values and are less sensitive to small biases (*8*). Its strength is in the detection of larger errors.

One of the biggest advantages of using patient-based real-time quality control is the assured commutability of the results (*5*, *6*). Additionally, it also does not carry consumable costs and utilizes the data already generated from clinical testing. As its name implies, it also provides continuous feedback on the analytical performance of the device each time a new result is generated. There are several limitations to adopting the MovSum and MA techniques at present. Firstly, these techniques are not yet routinely available in the glucometer software or middleware (*13*). As such, for routine application, it is necessary to separately extract the laboratory data from the middleware and exporting it to specialised statistical software for analysis. Such analysis requires familiarity with the statistical concepts for optimal performance, and requires additional time and human resource. Additionally, the optimisation of these techniques on multiple devices have not been assessed, and is an important area of future research.

In conclusion, this study has demonstrated the potential use of patient-based real-time quality control in point of care glucose meter. It provides an independent indicator of the instrument performance that is notsusceptible to the occasional QC failure due to operator error or material instability. The MovSum technique is suited for detecting small but clinically significant biases. Point of care QC should follow conventional practice by setting the control limits according to the running mean and SD to allow proper error detection. The glucometer manufacturers have an active role to play in liberalizing QC settings and also enhancing the middleware to facility patient-based QC practices.
